# Role of time delay on intracellular calcium dynamics driven by non-Gaussian noises

**DOI:** 10.1038/srep25067

**Published:** 2016-04-28

**Authors:** Wei-Long Duan, Chunhua Zeng

**Affiliations:** 1 City college/State Key Laboratory of Complex Nonferrous Metal Resources Clean Utilization, Kunming University of Science and Technology, Kunming 650051, P.R. China; 2 Faculty of Science, Kunming University of Science and Technology, Kunming 650093, P.R. China

## Abstract

Effect of time delay (*τ*) on intracellular calcium dynamics with non-Gaussian noises in transmission processes of intracellular Ca^2+^ is studied by means of second-order stochastic Runge-Kutta type algorithm. By simulating and analyzing time series, normalized autocorrelation function, and characteristic correlation time of cytosolic and calcium store’s Ca^2+^ concentration, the results exhibit: (i) intracellular calcium dynamics’s time coherence disappears and stability strengthens as *τ* → 0.1s; (ii) for the case of *τ* < 0.1s, the normalized autocorrelation functions of cytosolic and calcium store’s Ca^2+^ concentration show damped motion when *τ* is very short, but they trend to a level line as *τ* → 0.1s, and for the case of *τ* > 0.1s, they show different variation as *τ* increases, the former changes from underdamped motion to a level line, but the latter changes from damped motion to underdamped motion; and (iii) at the moderate value of time delay, reverse resonance occurs both in cytosol and calcium store.

Ca^2+^ is an ubiquitous and versatile second messenger that transmits information through changing the cytosolic Ca^2+^ concentration, namely, Ca^2+^ signaling pathway translates external signals into intracellular responses by increasing the cytosolic Ca^2+^ concentration in a stimulus dependent pattern. Ca^2+^ is mostly stored in the endoplasmic reticulum and mitochondria, namely calcium store. Specifically, the increasing of concentration can be caused either by Ca^2+^ entry from the extracellular medium through plasma membrane channels, or by Ca^2+^ release from the internal calcium store. This is the well-known calcium-induced calcium release mechanism.

In many studies on intracellular calcium oscillation(ICO) system, there have been a variety of channels showing calcium-induced calcium release and a variety of models^1^,^2^,^3^,^4^, and some phenomena have been found. Such as stochastic resonance in mesoscopic stochastic model^5^ and in ICO with Gaussian white noises and time delay^6^, reverse resonance in ICO with Gaussian colored noises and time delay^6^ and in ICO with non-Gaussian noises^7^, coherence resonance in ICO with non-Gaussian noises^7^ and in ICO with internal and external noises^8^, oscillatory coherence^9^ and resonant activation^10^ in ICO with Gaussian colored noises and time delay. Besides, calcium puffs has been found in neuronal cells^11^ and in a model with clustered Ca^2+^ release channels^12^, where, local waves, abortive waves, global oscillation and tide waves also have been exhibited. For other phenomena, there are stochastic backfiring in stochastic DeYoung-Keizer-model of the inositol 1,4,5-trisphosphate receptor channel^13^, dispersion gap and localized spiral waves in a bistable three component reaction-diffusion system modeling ICO^14^, Ca^2+^ spiral wave based on modified spatially extended Tang-Othmer Ca^2+^ model^15^.

In ICO system, it is inevitable to have the effect of stochastic force, i.e., noise. Therefore, Matjaž Perc group^16^,^17^,^18^,^19^,^20^,^21^,^22^ has studied the effects of noise on ICO. By analyzing experimental data, they have derived directly that noise and other stochastic effects indeed play a central role^16^,^17^. At the same time, they have obtained that, noise is good for the stability and robustness of ICO^18^ and for detecting of weak calcium signals within the cell^19^, and noise could induce periodic calcium waves^20^,^21^. When the cells are coupled, the effects of noise on ICO become lesser^22^. Then, they have introduced this spatial coherence resonance to excitable media^23^,^24^.

Martin Falcke group^13^,^14^,^25^,^26^,^27^,^28^,^29^,^30^,^31^,^32^,^33^,^34^,^35^ has done much research about ICO. They have studied a discrete stochastic model for calcium dynamics in living cells^25^, spatial and temporal structures in intracellular Ca^2+^ dynamics caused by fuctuations^26^, and key characteristics of Ca^2+^ puffs in deterministic and stochastic frameworks^30^.

In our research on ICO^6^,^7^,^9^,^10^,^36^,^37^,^38^,^39^,^40^,^41^, taking into account time delay in processes of active and passive transport of intracellular Ca^2+^, the roles of time delay and Gaussian colored noises on ICO system have been studied. In view of non-Gaussian noise in ICO with time delay, we have only studied the role of non-Gaussian noises on ICO, but the effect of time delay on ICO system has not been studied. Thus, studying the effect of time delay on ICO system with non-Gaussian noises, specifically, studying the effect of time delay on autocorrelation property of this ICO system is necessary in this paper.

First, according to ref. 7, the ICO model with non-Gaussian noises and time delay is presented. Then, the time delay, normalized autocorrelation function(NAF), and characteristic correlation time(CCT) of cytosolic and calcium store’s Ca^2+^ concentration are respectively simulated. Finally, conclusions are drawn.

## The model for ICO with non-Gaussian noises

There is a layer of membrane to separate calcium store and cytosol, in the membrane there is some channel cluster. In transport process of intracellular Ca^2+^ between cytosol and calcium store, it depends that channel cluster opens as transport pathway, in this way Ca^2+^ could only transport from cytosol into calcium store or from calcium store into cytosol. When Ca^2+^ transporting in channel, no matter what from cytosol into calcium store, or from calcium store into cytosol, it is sure that this process all takes time. Moreover, both the transport process from cytosol into calcium store and the transport process from calcium store into cytosol, they all contain two processes, i.e., the active transport process and the passive transport process. In order to study easily, taking into account same time delay *τ* in processes of active and passive transport of Ca^2+^ in a real cell. In this paper, *x* and *y* denote the concentration of free Ca^2+^ of cytosol and calcium store in a cell, respectively. Based on calcium-induced calcium release, the Langevin equations of ICO system can be read as follows according to our previous result^7^:


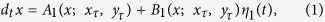



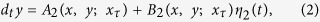


with


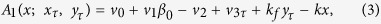



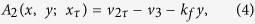



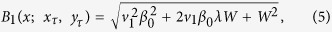



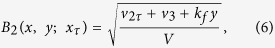



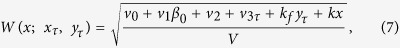


and


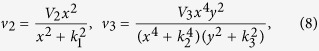



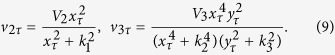


Here *v*_0_ is the steady flow of Ca^2+^ to the cytosol, *v*_1_ is the maximum rate of the stimulus induced influx of Ca^2+^ from the extracellular medium, *β*_0_ is the external control parameter that denotes the degree of extracellular simulation. The rates *v*_2_ and *v*_3_ refer, respectively, to pumping of Ca^2+^ into calcium store and to release of Ca^2+^ from store into cytosol in a process activated by cytosolic Ca^2+^. *v*_2*τ*_ is *v*_2_ with time delay, and *v*_3*τ*_ is *v*_3_ with time delay. *k*_*f*_*y* is a diffusional flow of Ca^2+^ from store to cytosol, *kx* denotes the uptake from the cytosol, *V* is the system size. *V*_2_ and *V*_3_ denote the maximum values of the rates *v*_2_ and *v*_3_, respectively. The parameters *k*_1_, *k*_2_, and *k*_3_ are threshold constants for pumping, release, and activation of release by Ca^2+^ and by inositol 1,4,5-trisphosphate. *W* = *W*(*x*; *x*_*τ*_, *y*_*τ*_), *x*_*τ*_ = *x*(*t* − *τ*), *y*_*τ*_ = *y*(*t* − *τ*). *λ* denotes cross-correlation degree of internal and external noise before merger^38^.

By analysising experimental data, Matjaž Perc group^16^ have established that the nature of ICO is stochastic. Specifically, namely it is noise to act on the transport process of intracellular Ca^2+^ between cytosol and calcium store. Based on stochastic dynamics, the noise is divided into Gaussian noise and non-Gaussian noise. Among the Gaussian noise only exists in stochastic dynamics system under the ideal process, but the noise in the really stochastic dynamics system, including biological system, is basically all non-Gaussian noise, which also contains Gaussian noise only if the control parameter takes 1 then non-Gaussian noise evolves into Gaussian noise. Importantly, after careful analysising experimental data and comparing with mathematical models of Martin Falcke group’s results^35^, we conclude, the noise in real ICO is very likely non-Gaussian noise. So that the noises *η*_1_(*t*) and *η*_2_(*t*) in Eqs (1 and 2) are considered as non-Gaussian noises which are characterized by the following Langevin equation^42^:


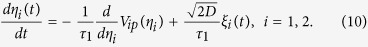


Where *ξ*_*i*_(*t*) is a standard Gaussian white noise of zero mean and correlation *ξ*_*i*_(*t*)*ξ*_*i*_(*t*′) = *δ*(*t* − *t*′). *V*_*ip*_(*η*_*i*_) is given by


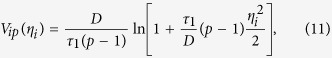


and the statistical properties of non-Gaussian noise *η*_*i*_(*t*) is defined as


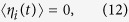



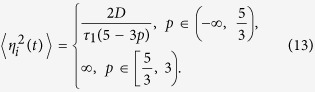


Where *τ*_1_ denotes the correlation time of the non-Gaussian noises *η*_*i*_(*t*), and *D* denotes the noise intensity of Gaussian white noise *ξ*_*i*_(*t*). The parameter *p* is used to control the degree of the departure from the non-Gaussian noise to Gaussian noise. The distribution of the noise is Gaussian for *p* = 1, non-Gaussian with long tail for *p* > 1, and characterized by a “more than Gaussian” cutoff for *p* < 1. Here, in order to study easily, supposing noises *ξ*_1_(*t*) and *ξ*_2_(*t*) have same strength *D*, and non-Gaussian noises *η*_1_(*t*) and *η*_2_(*t*) have same *p* and correlation time *τ*_1_.

## Time series, NAF and CCT of intracellular Ca^2+^ concentration

By means of second-order stochastic Runge-Kutta type algorithm^43^, for the specific simulation algorithm of ICO system, see ref. 7, discretize time in steps of size Δ = 0.001s, one can stochastically simulate the time evolution of intracellular Ca^2+^ concentration in the cytosol *x*(*t*) and calcium store *y*(*t*). Experimentally, *x* is in the order of 100~200 nM in basal state^44^ and *y* = 5 *μ*M^45^, so that the initial values *x*(0) and *y*(0) independently take uniformly random from 0.1~0.2 *μ*M and 4~5 *μ*M. In the condition of time delay, it is rational to let *x*(*t* − *τ*) = *x*(0) and *y*(*t* − *τ*) = *y*(0) as *t* < *τ*. The value of parameters are set as^7^: *v*_0_ = 1 *μ*M/s, *v*_1_ = 7.3 *μ*M/s, *β*_0_ = 0.287, *k*_*f*_ = 1/s, *k* = 10/s, *V*_2_ = 65 *μ*M/s, *V*_3_ = 500 *μ*M/s, *k*_1_ = 1 *μ*M, *k*_2_ = 0.9 *μ*M, *k*_3_ = 2 *μ*M, *V* = 1000 *μ*m^3^, and *λ* = 0.1. Additionally, in this paper the parameters of non-Gaussian noises are *p* = 0.9, *D* = 0.5, and *τ*_1_ = 10 s.

**Figure 1:**
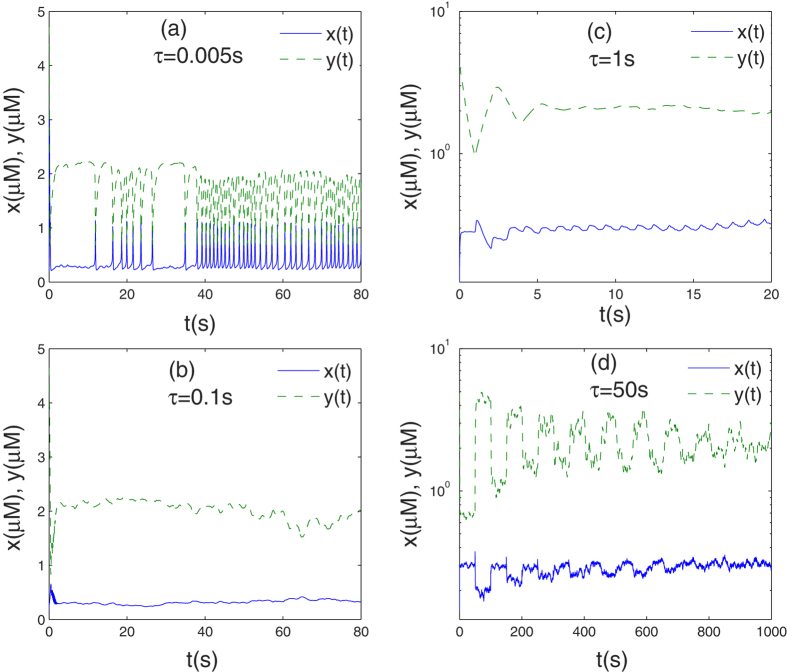
The variation of time series of Ca^2+^ concentration in cytosol *x*(*t*)(solid line) and calcium store *y*(*t*)(dotted line) for time delay *τ*.

First, the variation of time series for time delay is plotted in Fig. 1. There is also a critical value *τ*_*c*_ ≃ 0.1 s of *τ*. As *τ* → *τ*_*c*_, ICO almost disappears(see Fig. 1(b), *τ* = 0.1s). Either *τ* < *τ*_*c*_, the time coherence is obvious when time delay *τ* is short(see Fig. 1(a), *τ* = 0.005s), or *τ* > *τ*_*c*_, the oscillation also strengthens as time delay increases(see Fig. 1(c–d), *τ* = 1 s and 50 s). In addition, it is clearly seen that Ca^2+^ concentrations in cytosol *x*(*t*) is lower than calcium store *y*(*t*), this is also fact in a real cell. By simulating time series, it could obtains many properties of stochastic dynamics system, e.g., in neuronal network system^46^,^47^,^48^.

Second, to describe the fluctuation decay of intracellular Ca^2+^ concentration of cytosol *x*(*t*) and calcium store *y*(*t*) in the stationary state, one respectively define NAF of state variable *x*(*t*) and *y*(*t*) as *C*_*x*_(*θ*) and *C*_*y*_(*θ*)(see refs 7 and 9) as follows


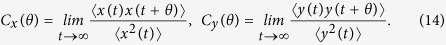


Where *θ* is autocorrelation time, point bracket 〈〉 denotes statistical average over time.

**Figure 2:**
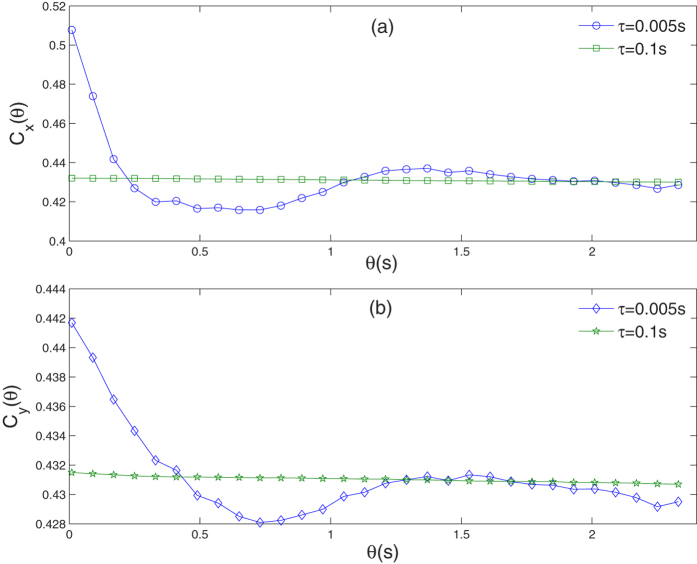
For the case of short time delay, the role of time delay on NAFs vs. autocorrelation time *θ* is simulated.

**Figure 3:**
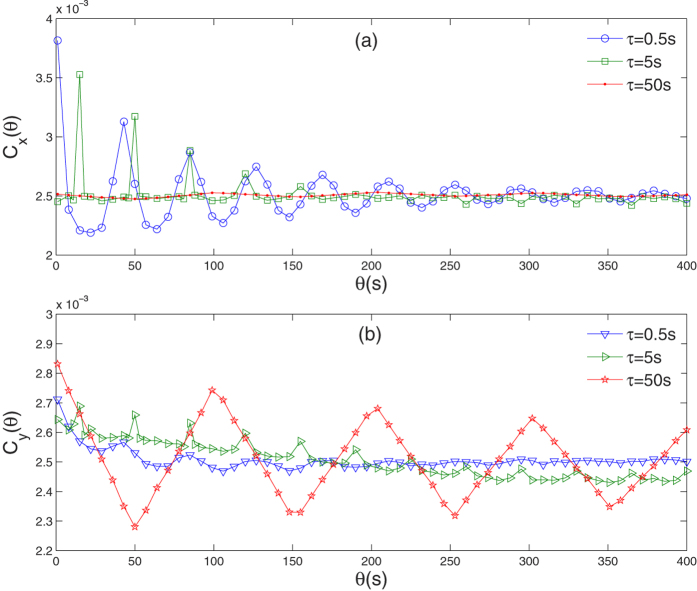
For the case of long time delay, the role of time delay on NAFs vs. autocorrelation time *θ* is simulated.

*C*_*x*_(*θ*) and *C*_*y*_(*θ*) vs. autocorrelation time *θ* are studied as time delay varies. In Fig. 2, for the case of short time delay, both *C*_*x*_(*θ*) and *C*_*y*_(*θ*) have similar regular with time delay, i.e., both NAFs show damped motion when time delay is very short(e.g., *τ* = 0.005s), as time delay increases, however, they become a level line(e.g., *τ* = 0.1s). In Fig. 3, for the case of long time delay, where NAFs *C*_*x*_(*θ*) and *C*_*y*_(*θ*) have different regular: in Fig. 3(a), *C*_*x*_(*θ*) exhibits underdamped motion with moderate time delay(e.g., *τ* = 0.5 s), and it is almost periodic motion, but this motion slowly decreases as time delay prolongs(e.g., *τ* = 5 s), finally it becomes a level line(e.g., *τ* = 50 s); in Fig. 3(b), *C*_*y*_(*θ*) exhibits damped motion with moderate time delay(e.g., *τ* = 0.5 s), but it finally becomes underdamped motion(e.g., *τ* = 50 s), and it is almost periodic motion.

In order to explain the ordering of time series, it is necessary to introduce CCT Λ_*x*_ of cytosolic Ca^2+^ concentration *x*(*t*) and Λ_*y*_ of calcium store’s Ca^2+^ concentration *y*(*t*) to reflect correlation, they can be defined as follows


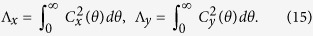


The larger the CCT, the more pronounced the correlation is, and the more orderly the time series is.

**Figure 4:**
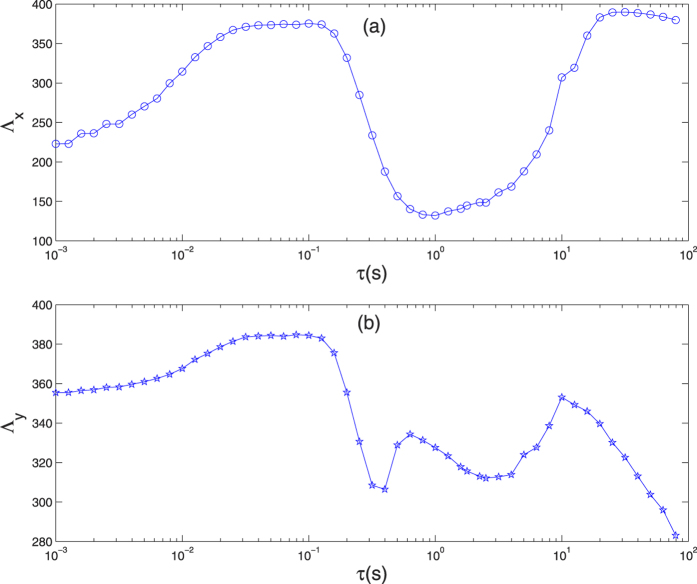
CCT Λ_*x*_ and Λ_*y*_ vs. time delay are simulated.

In Fig. 4, Λ_*x*_ and Λ_*y*_ vs. time delay *τ* is plotted. Here, CCTs Λ_*x*_ and Λ_*y*_ have different regular as time delay varies. In Fig. 4(a), the variation of CCT Λ_*x*_ for time delay shows, it firstly increases and then exhibits a concave structure, which implies that the moderate time delay induces reverse resonance in cytosolic calcium oscillation. In Fig. 4(b), the variation of CCT Λ_*y*_ for time delay shows, it firstly increases and then presents double concave structure, which also implies that the moderate time delay induces reverse resonance in calcium store’s calcium oscillation. Anyway, these manifest a reverse resonance phenomenon with respect to time delay in ICO system with non-Gaussian noises.

Among our previous research, when we studying the role of time delay on ICO, there is a different phenomena with different type noise. If noise is Gaussian white noise, there is also critical phenomenon of time delay^37^,^39^. If noise is Gaussian colored noise, time delay would induce reverse resonance and stochastic resonance^6^, oscillatory coherence^9^, resonant activation^10^, periodic square calcium wave^36^, stability transition^40^, calcium spikes^41^. Comparing above results concerning non-Gaussian noise with previous our works about Gaussian one, there is many differences. In the other stochastic system with time delay^49^,^50^,^51^,^52^,^53^, one have also researched about the role of time delay.

## Conclusions

In view of non-Gaussian noises and time delay in transmission processes of intracellular Ca^2+^, by means of second-order stochastic Runge-Kutta type algorithm, we have studied the role of time delay on intracellular calcium dynamics. By computing the time series, NAF, and CCT of cytosolic and calcium store’s Ca^2+^ concentration, some conclusions are obtained.

Firstly, the effects of time delay on time series are analyzed, there is a critical value of 0.1s of time delay: ICO decreases as time delay trends to this value, however, ICO is very obvious as time delay doesn’t trend to this value. Especially, time coherence appears when time delay is very short. Then, the variation of NAF for time delay shows: for the case of short time delay, both NAFs of cytosolic and calcium store’s Ca^2+^ concentration present damped motion when time delay is very short, but finally they become a level line as time delay increases. For the case of long time delay, NAF of cytosolic Ca^2+^ concentration presents underdamped motion, but it decreases into a level line as time delay further prolongs; NAF of calcium store’s Ca^2+^ concentration changes from damped motion to underdamped motion as time delay further prolongs. Finally, the variation of CCT for time delay shows: CCT of cytosolic and calcium store’s Ca^2+^ concentration respectively present a concave structure and two concave structure as time delay varies, these imply that reverse resonance occurs both in cytosol and calcium store when ICO system is driven non-Gaussian noises and time delay.

## Additional Information

**How to cite this article**: Duan, W.-L. and Zeng, C. Role of time delay on intracellular calcium dynamics driven by non-Gaussian noises. *Sci. Rep.* **6**, 25067; doi: 10.1038/srep25067 (2016).
